# Dose-dependent protective effect of nicotine in a murine model of viral myocarditis induced by coxsackievirus B3

**DOI:** 10.1038/srep15895

**Published:** 2015-10-28

**Authors:** Ge Li-Sha, Zhao Jing-Lin, Chen Guang-Yi, Liu Li, Zhou De-Pu, Li Yue-Chun

**Affiliations:** 1 Department of Pediatrics, Second Affiliated Hospital of Wenzhou Medical University, Wenzhou 325000, China; 2 Department of Cardiology, Second Affiliated Hospital of Wenzhou Medical University, Wenzhou 325000, China

## Abstract

The alpha 7 nicotinic acetylcholine receptor (alpha7 nAChR) was recently described as an anti-inflammatory target in various inflammatory diseases. The aim of this study was to investigate the dose-related effects of nicotine, an alpha7 nAChR agonist, in murine model of viral myocarditis. BALB/C mice were infected by an intraperitoneally injection with coxsackievirus B3. Nicotine was administered at doses of 0.1, 0.2 or 0.4 mg/kg three times per day for 7 or 14 consecutive days. The effects of nicotine on survival, myocardial histopathological changes, cardiac function, and cytokine levels were studied. The survival rate on day 14 increased in a dose-dependent fashion and was markedly higher in the 0.2 and 0.4 mg/kg nicotine groups than in the infected untreated group. Treatment with high-dose nicotine reduced the myocardial inflammation and improved the impaired left ventricular function in infected mice. The mRNA expressions and protein levels of TNF-*α*, IL-1*β*, IL-6, and IL-17A were significantly downregulated in dose-dependent manners in the nicotine treatment groups compared to the infected untreated group. Nicotine dose-dependently reduced the severity of viral myocarditis through inhibiting the production of proinflammatory cytokines. The findings suggest that alpha7 nAChR agonists may be a promising new strategy for patients with viral myocarditis.

Viral myocarditis is an inflammatory heart disease that involves the myocardium or heart muscle caused by cardiotropic virus infection, and has been identified as an important cause of heart failure and dilated cardiomyopathy, especially in young patients^1^. The common infectious trigger of viral myocarditis is believed to be the enteroviruses of the picornavirus family, such as the coxsackie B virus group^1^. In spite of decades of extensive effort, including common or novel immunization procedures in animal models and clinical trials, no virus-specific preventive measures against coxsackievirus B3 (CVB3)-induced myocarditis are currently in clinical use^2^,^3^. Although the mechanisms involved in the pathogenesis of viral myocarditis are not well understood, cytokine-associated damage to myocytes has been demonstrated^4^,^5^,^6^. It has been suggested that interleukin (IL)-1*β*, IL-6 and tumour necrosis factor (TNF)-*α* play an essential role in the pathophysiology of murine viral myocarditis and that the suppression of IL-1*β*, IL-6 and TNF-*α* can moderate acute myocarditis^4^,^5^,^6^. In the early phase, viruses that evade the innate immune system replicate, producing viral proteins that infect the myocardium and cause direct myocyte damage and dysfunction. The early phase often passes unnoticed because the direct viral injury is frequently prevented by the innate immune response. The intermediate and final phases develop because of the immune dysregulation that is triggered by the initial cardiac injury^1^. The innate immune response has a crucial role for host defense during the infection^7^. The early inflammatory cytokine imbalance is important. Innate immune cytokines such as TNF-*α*, IL-1*β*, and IL-6 are essential for the development of acute viral-induced myocarditis. The expressions of cytokine genomic RNA, particularly TNF-*α*, IL-1*β*, and IL-6 are upregulated in viral myocarditis and might be induced by infection of coxsackievirus, which indicates that proinflammatory cytokines play an important role in the development of viral-induced myocarditis^4^,^5^,^6^.

Recently, Tracey and other researchers have identified the “cholinergic anti-inflammatory pathway” as a mechanism for the neuronal control of inflammation through the vagal efferent nerve^8^. The cholinergic antiinflammatory pathway exerts antiinflammatory effects through the alpha 7 nicotinic acetylcholine receptor (alpha7 nAChRs), which is expressed not only by the specific cells in central nervous system but also by immune cells like CD4^+^ T cells and macrophages^9^,^10^,^11^,^12^. The signalling of acetylcholine through alpha7 nAChRs has been shown to reduce the levels of TNF-*α*, IL-1*β*, and IL-6^13^. Previous studies have indicated that selective alpha7 nAChR agonists improve long-term survival following chronic heart failure and improve the survival of mice with endotoxemia and severe sepsis^14^,^15^. In addition to inflammatory bowel disease, including ulcerative colitis and postoperative ileus, many clinical trials have also investigated the roles and contributions of alpha7 nAChR agonists in neurodegenerative diseases and other forms of dementia^16^,^17^,^18^. Leib *et al.* reported that nicotine, an alpha7 nAChR agonists, decreased heart inflammation in a murine autoimmune myocarditis model^19^. Recently, we have also found that activation of the cholinergic anti-inflammatory pathway reduced inflammation in viral myocarditis^20^. However, the dose-dependent effects of alpha7 nAChR agonists in acute murine viral-induced myocarditis and on cytokine production are not well known. The present study was performed to examine the therapeutic effects of different doses of the selective alpha7 nAChR agonist nicotine in a murine model of viral myocarditis induced by CVB3 infection.

## Results

### Effects of nicotine treatment on myocardial histopathology on days 7 and 14

**Figure 1:**
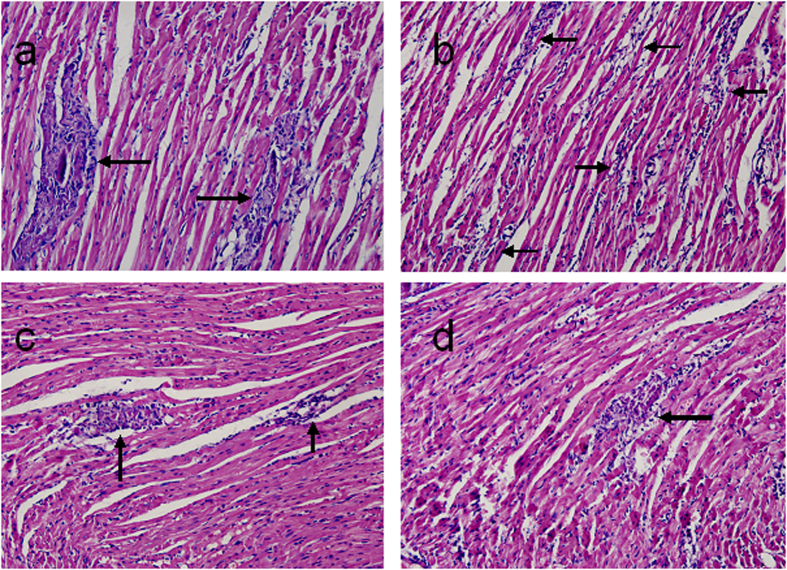
Histopathology in the heart on day 7 (Hematoxylin Eosin × 200). (**A**) Representative histopathology of the myocarditis group. There are two large foci of inflammatory cellular infiltration (arrow) found in the region (Infiltration score: 2.5; Necrosis score: 1.8). (**B**) Representative histopathology of the mice treated with 0.1 mg/kg nicotine. There are several small foci of inflammatory cellular infiltration (arrow) found in the region (Infiltration score: 2.1; Necrosis score: 1.5). (**C**) Representative histopathology of the mice treated with 0.2 mg/kg nicotine. There are two small foci of inflammatory cellular infiltration (arrow) found in the region (Infiltration score: 1.4; Necrosis score:1.2). (**D**) Representative histopathology of the mice treated with 0.4 mg/kg nicotin. There is a small foci of inflammatory cellular infiltration (arrow) found in the region (Infiltration score: 1.2; Necrosis score: 0.9).

Upon the sacrifices that were performed on days 7 and 14, severe injuries to the myocardia and cellular infiltration were found in the myocarditis group. Significant reductions in the cardiac pathological scores, including infiltration and necrosis were achieved in the groups with high- and medium-dose nicotine treatments compared with the myocarditis group (i.e., the infected untreated group) (Fig. 1, Table 1).

### Effects of nicotine treatment on survival rate

**Figure 2:**
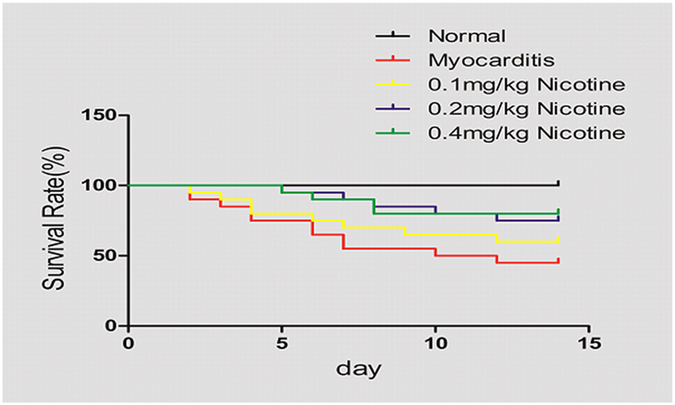
Survival rates to day 14 (n = 10 in each group). The survival rates of the CVB3-infected mice were followed for 14 days and were 100% for the normal control group, 45.0% for the myocarditis group (i.e., the infected untreated group), 80.0% for the mice treated with 0.4 mg/kg nicotine, 75% for the mice treated with 0.2 mg/kg, and 60% for the mice treated with 0.1 mg/kg. The experiment was done 3 times.

The mice that were inoculated with CVB3 exhibited the virus infection syndrome, which included as loose hair, being idle, poor appetite and reduced body weight, from day 3. Deaths peaked between days 5 and 10. Compared to the mice in the myocarditis group, the high- and medium-dose nicotine treatments significantly decreased mouse mortality (P < 0.05) and increased the survival rate as shown in Fig. 2. The survival rate of the low-dose nicotine treatment group did not differ compared from that of the myocarditis group (P > 0.05).

### Viral replication in the Myocardium

**Figure 3:**
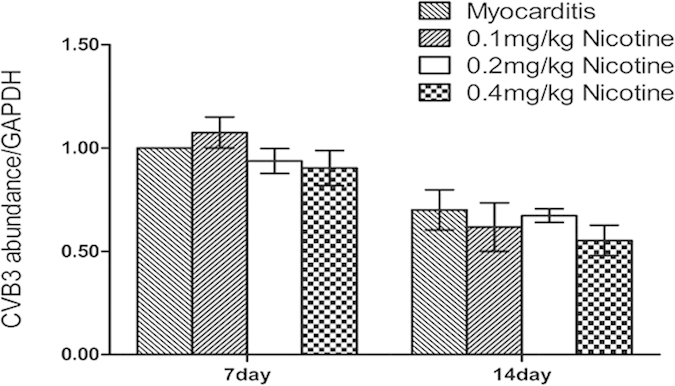
Expression of CVB3 mRNAs by quantitative PCR analysis in the myocardial tissues of mice on days 7 and 14 (n = 8 in each group). The experiment was repeated 3 times.

Fluorescent quantitative PCR-analysis showed CVB3-RNA abundance in the myocardium of the infected mice on days 7 and 14. No significant differences were found in the abundances of CVB3-RNA between the treatment groups and the myocarditis group on days 7 or 14 (Fig. 3).

### Echocardiographic findings on day 14

**Figure 4:**
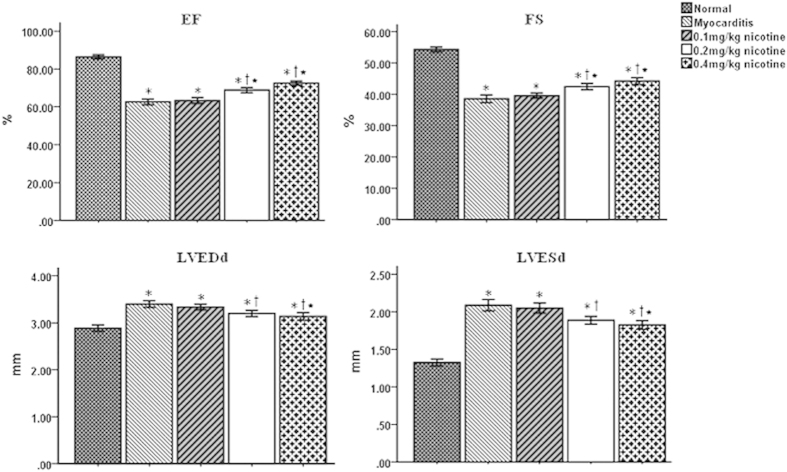
Echocardiographic results on day 14 (n = 8 in each group). EF, left ventricular ejection fraction; FS: fractional shortening; LVEDd, left ventricular end-diastolic diameter; LVESd, left ventricular end-systolic diameter; mm, millimetre. *P < 0.05 versus normal; ^†^P < 0.05 versus myocarditis; *P < 0.05 versus 0.1 mg/kg nicotine group.

The left ventricular ejection fractions (EFs) and fractional shortenings (FSs) of the CVB3- inoculated groups were significantly decreased compared to those of the normal control group on day 14. The EFs and FSs of the 0.2 and 0.4 mg/kg nicotine groups were significantly increased compared to those of the 0.1 mg/kg nicotine group and the myocarditis group on day 14, but the EFs and FSs of the 0.1 mg/kg nicotine group did not differ from those of the myocarditis group (Fig. 4). The left ventricular end-diastolic internal diameters (LVEDds) and left ventricular end-systolic internal diameters (LVESds) of the CVB3-infected groups were increased compared to those of the normal control group on day 14 (Fig. 4). The LVEDds and LVESds of the 0.4 mg/kg nicotine group were significantly reduced compared to those of the 0.1 mg/kg nicotine and myocarditis groups on day 14, but the LVEDds and LVESds of the 0.1 mg/kg nicotine group did not differ from those of the myocarditis or 0.2 mg/kg nicotine groups (Fig. 4).

### Cytokine Gene Expression in the Heart

**Figure 5:**
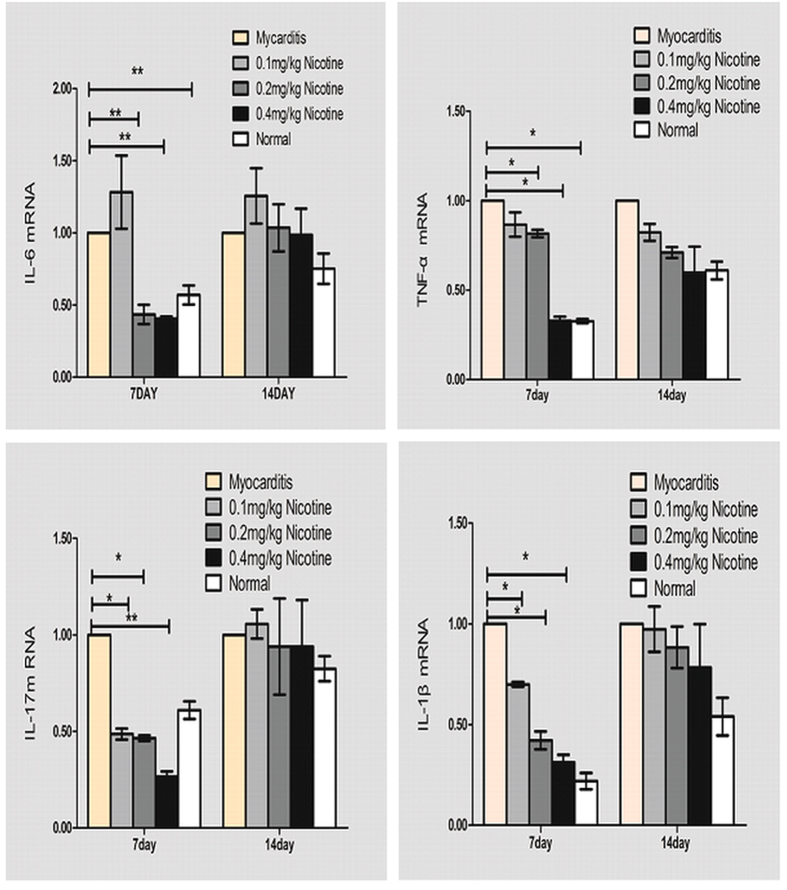
Expression of cytokine mRNAs in the myocardial tissues of mice on days 7 and 14 (n = 8 in each group). *P < 0.05 versus normal; ^†^P < 0.05 versus myocarditis; ^★^P < 0.05 versus 0.1 mg/kg nicotine group. The experiment was repeated 3 times.

On days 7 and 14, the mRNA production of TNF-*α*, IL-6, IL-17A and IL-1*β* in the myocardia of the CVB3-inoculated mice were significantly increased compared to levels observed in the normal group (Fig. 5). On day 7, the cardiac IL-1*β*, TNF-*α*, IL-6 and IL-17A levels were significantly downregulated in the group of 0.2 and 0.4 mg/kg nicotine compared to the myocarditis group. On day 7, the mRNA levels of TNF-*α*, IL-1*β* and IL-17A were reduced in the group of 0.1 mg/kg nicotine compared to the myocarditis group. However, the IL-6 mRNA levels were significantly higher in the 0.1 mg/kg nicotine group than in the myocarditis group. On day 14, There is no differences in the TNF-*α*, IL-1*β*, IL-6 or IL-17A mRNA levels between the myocarditis group and the treatment groups.

### ELISA Analyses of Cytokine Levels in the Heart

**Figure 6:**
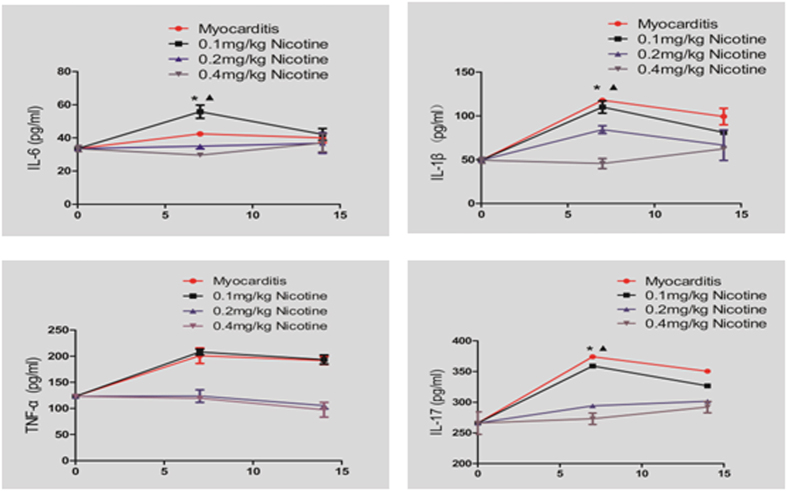
Expressions of cytokines in the myocardial tissues of the mice on days 7 and 14 (n = 8 in each group). ^†^P < 0.05 versus myocarditis; ^★^P < 0.05 versus 0.1 mg/kg nicotine group. The experiment was repeated 3 times.

Compared to the myocarditis group, the levels of TNF-*α*, IL-1*β* and IL-17A were significantly reduced in the treatment groups, but the levels of IL-6 were increased in the 0.1 mg/kg nicotine group on day 7 (Fig. 6). On day 14, there were no significant differences in the cytokine levels between the treatment groups and the myocarditis group.

## Discussion

### Major findings

In this research, we examined the therapeutic effects of nicotine in viral myocarditis. Our results indicate that stimulation of alpha7 nAChRs with nicotine significantly reduced the severity of acute myocarditis induced by CVB3 in mice. Nicotine treatment improved the 14-day survival of the infected mice in a dose-dependent manner. The high- and medium-dose nicotine treatments ameliorated the myocardial lesions and improved the impairments of left ventricular function of mice infected with CVB3. The myocardial expressions of cytokine (IL-1*β*, IL-6, IL-17A and TNF-*α*) mRNAs were reduced in the high- and medium-dose nicotine-treated mice. Moreover, the protein levels of the proinflammatory cytokines IL-1*β*, TNF-*α*, IL-6 and IL-17A were reduced in the mice that were with high- and medium-dose nicotine. Notably the low-dose nicotine (in contrast to the high-dose nicotine) was not effective when administered for up to 7 days, which indicates a dose-dependent effect of nicotine treatment. These data show that the short-term activation of alpha7 nAChRs is sufficient to halt the progression of myocarditis when the nicotine concentration is sufficiently high. To the best of our knowledge, this is the first study to investigate the dose-related anti-inflammatory effects of nicotine in viral myocarditis.

### Therapeutic effects of different doses of nicotine in viral myocarditis

The experimental evidence that has accumulated over the last few years has demonstrated that the activation of the cholinergic anti-inflammatory pathway, either via direct activation of nicotinic acetylcholine receptors by nicotine administration or vagal stimulation, reduces secretion of the proinflammatory cytokines TNF-*α*, IL-1*β*, and IL-6 in experimental models of acute systemic inflammation. The selective alpha7 nAChR agonist GTS-21 dose-dependently inhibits the production of TNF-*α* and improves survival during endotoxemia^15^. Stimulation of alpha7 nAChRs with nicotine attenuates the inflammatory responses in macrophages through heme oxygenase-1 upregulation, and dose- and time-dependently improves survival in an experimental model of sepsis^21^. The acetylcholinesterase inhibitor galantamine dose-dependently suppresses serum TNF-*α* in endotoxemic mice^22^. Borovikov *et al.* found that acetylcholine dose-dependently inhibits the production of TNF-*α* in macrophage cultures conditioned by exposure to lipopolysaccharide for 4 h^8^. Nicotine significantly blocks the TNF-*α*-induced expression of adhesion molecules and chemokines in human microvascular endothelial cells in dose dependent manners^23^. These findings suggest that alpha7 nAChR agonists dose-dependently inhibit inflammation via the cholinergic anti-inflammatory pathway. Our results indicate that stimulation of alpha7 nAChRs with nicotine treatment dose-dependently improved survival, reduced myocardial inflammation and improved the impairment of left ventricular function of mice infected by CVB3.

### Effects of different doses of nicotine on myocardial cytokines

Activation of the sympathetic nervous system in myocarditis has been studied^24^,^25^,^26^,^27^,^28^. Studies from our group and other groups have recently demonstrated that the levels of plasma epinephrine and noradrenaline and cardiac sympathetic nerve activity are increased in myocarditis, and the non-selective *β*-blocker carvedilol reduces the production of proinflammatory cytokines and plasma epinephrine and noradrenaline and ameliorates murine viral myocarditis^24^,^25^,^26^,^27^,^28^. In the present study, treatment with 0.2 mg/kg and 0.4 mg/kg nicotine significantly suppressed the release of inflammatory cytokines and the associated inflammation. These findings suggest that autonomic nervous system dysfunction (sympathetic activation and vagal withdrawal) might play a crucial role in murine viral myocarditis. Nicotine has been shown to attenuate the release of the proinflammatory cytokines in various models of inflammation, including sepsis, ischemia,reperfusion, haemorrhage, endotoxemia, postoperative ileus and heart failure^16^,^17^,^18^. A recent study by Leib *et al.* has shown that the administration of nicotine can reduce the expression of TNF-*α* and IL-6, and decreased the production of monocyte chemoattractant protein-1 and macrophage inflammatory protein-1 in a murine autoimmune myocarditis model^19^. In support of these findings, the myocardial mRNA and protein expressions of the proinflammatory cytokines IL-1*β*, IL-6, TNF-*α* and IL-17A were dose-dependently reduced in CVB3-infected mice that were treated with nicotine in the present study. It has been suggested that proinflammatory cytokines play an important roles in the pathophysiology of myocarditis and that the suppression of these proinflammatory cytokines can moderate acute myocarditis^7^. Therefore, stimulation of alpha7 nAChRs with nicotine has protective effects against viral myocarditis.

The differences in cytokine levels (mRNA and protein) were statistically significant in the CVB3-infected mice, but they were numerically small in the present study, which indicates that inflammatory cytokines are important for regulating the severity of inflammation. Proinflammatory cytokines such as TNF-*α*, IL-1*β*, and IL-6 have been functionally studied as treatment targets in the context of viral myocarditis; their effects are often dose- and time-dependent^4^,^5^,^6^,^29^,^30^,^31^. Either insufficient or exaggerated expression of TNF-*α* and IL-6 is equally harmful^29^,^30^,^31^. Small but significant changes of cytokine levels after treament may lead to significant difference in their response to treament^29^,^30^,^31^.

Acetylcholine, the principal vagal neurotransmitter, is the endogenous alpha7 nAChR agonist^32^. The neuronal source of cardiac acetylcholine is synthesized by the cardiac vagal neurons and stored in the vesicles of cholinergic fiber. Cardiomyocyte and vascular endothelial cell also can synthesize non-neuronal source of acetylcholine^33^,^34^,^35^. Therefore, it is likely that endogenous non-neuronal acetylcholine released from cardiomyocytes activates nAChRs. Signaling of acetylcholine through *α*7-nAChR has been shown to inhibit the production of TNF-*α*, IL-1*β*, IL-6 and IL-17^8^. It has been shown that nicotine, as a specific agonist of alpha7 nAChR, is more effective than acetylcholine at attenuating the production of the proinflammatory cytokines^36^. Previous studies demonstrated that nicotine was mimetic of vagus nerve stimulation, acting directly as an agonist on alpha7 nAChR^37^. However, other studies also found that nicotine might stimulate catecholamine release by an activation of nAChR localized on the adrenal medulla and peripheral postganglionic sympathetic nerve endings^38^. Therefore, there is a limitation to use the effects of nicotine presented in the study to support the statement that autonomic nervous system dysfunction (vagal withdrawal and sympathetic activation) might play a crucial part in murine viral myocarditis.

In conclusion, this study demonstates that nicotine has therapeutic benefits in murine CVB3-induced myocarditis and dose-dependently reduces the release of proinflammatory cytokines and inflammation. The findings suggest that alpha7 nAChR agonists may be a promising new strategy for patients with viral myocarditis. Although the beneficial effects of chemical vagal stimulation on myocarditis mice were shown in the present study, the safety and potential adverse effects of such treatment remains unclear. The appropriate protocol for this type of treatment also remains unsettled and should be investigated. Large-scale, long-term trials of vagal nerve stimulation in animal models of myocarditis are required to establish a therapeutic strategy based on the results of this study.

## Methods

### Mice

Male BALB/c mice (4 weeks) were purchased from Shanghai Laboratory Animal Center, China. All mice were maintained in the Wenzhou Medical University animal facilities under specific pathogen-free conditions. The study conformed with China Animal Protection Law. The Wenzhou Medical University Committee on Ethics in the Use and Care of Laboratory Animals approved the conduct of this study.

### Virus infection

Animals were infected as described in our previous publications^39^. Briefly, 1.0 × 10^6^ plaque forming units (pfu) of CVB3 (strain Nancy, ATCC VR-30) were diluted in 0.1 ml of phosphate-buffered saline and injected intraperitoneally. Control group was injected intraperitoneally with 0.1 ml of normal saline solution. We defined the day of virus inoculation as day 0.

### Drug Administration

Nicotine (product number: N73876-25ML, liquid form) was obtained from Sigma Aldrich Co. The times and doses were selected according to previous experiments^19^. Each animal was used for a single experiment. All drugs were freshly dissolved in saline immediately before use. Starting 24 h after infection, nicotine was injected intraperitoneally to the mice for 14 consecutive days. Groups of mice (n = 40 per group) were treated one of three doses of nicotine (0.1, 0.2, and 0.4 mg/kg) three times per day. The mice of normal group (n = 20) and mice of myocarditis (i.e., the infected untreated group, n = 40) received saline injections of equal volume. Eight mice from each group were sacrificed on days 7 and 14, blood samples were collected, and the hearts of mice were then removed and divided for histological and biochemical examinations. All mice were anesthetized with pentobarbital (100 mg/kg, one dose intraperitoneally) prior to sacrifice. Efficient anesthesia was monitored through pinching the hind paw, when sufficiently sedated the mice were euthanized through cervical dislocation.

### Echocardiographic examination

Transthoracic echocardiography was performed as described in our previous publications^24^. Each mouse were anesthetized with intraperitoneally with 3% chloral hydrate (0.01 ml/g) and was placed on its left side. The chest was shaved. An agilent Sonos 5500 ultrasound machine (Phillips, USA) equipped with a 12 MHz linear transducer, real time digital acquisition, storage, and review capabilities was used. M-mode, Two-dimensional, and Doppler flow images were obtained in parasternal long-axis view. The LVEDd and LVESd were measured over the course of at least 3 consecutive cardiac cycles. The EF and FS were then both calculated. The investigator who conducted the echocardiography was unknown of the treatment status of the respective mice.

### Myocardial Histopathology

The tissues of heart were fixed in 10% formaldehyde, and then embedded in paraffin. The sections were subsequently stained with hematoxylin and eosin. Two skilled observer who were blind to the experimental treatment scored for cellular infiltration and myocardial necrosis, as described in our previous publications^24^,^39^. The scores were as follows: 0 = no lesion; 1+ = lesions involving <25% of the myocardium; 2+ = lesions involving 25% to 50%; 3+ = lesions involving 50 to 75%; and 4+ = lesions involving 75% to 100%.

### Survival Rate

The survival was observed up to 14 days in the study.

### Quantitative Polymerase Chain Reaction

According to the manufacturer’s protocol (Invitrogen Corporation), total RNA was extracted from the heart tissue with the RNeasy Midi kit. cDNA synthesis (iScript cDNA Synthesis Kit, Fermentals corporation, Germany) was performed with 2 μg of RNA according to the manufacturer’s protocol. For the quantitative polymerase chain reaction, 1 μl of cDNA was used. After an initial denaturation of 95 °C for 30 seconds, 45 cycles were performed that consisted of denaturation at 95 °C for 5 seconds and annealing at 60 °C for 10 seconds. We used the primer sequences illustrated in Table 2 for the quantification of the transcript levels.

### Enzyme-linked immunosorbent assay for myocardial cytokines

Tissue piece of heart was weighed and then minced to small pieces, which will be homogenized in phosphate-buffered saline with a glass homogemizer on ice (100 mg tissue per ml of ice-cold homogenizer buffer). To further break the cells, the suspension was sonicated with an ulrasonic cell disrupter. The homogenates were then centrifugated for 20 minutes at 3000 *g* to get the supernate, which was used for subsequent determination of cytokine levels. Total protein concentration was assayed by Lowry method. The supernatants were diluted to the same concentration and aliquoted, and then stored at −80 °C until enzyme-linked immunosorbent assay (ELISA) analysis. The levels of IL-1*β*, IL-6, IL-17A and TNF-*α* were measured by a variety of ELISA kits according to the manufacturer’s protocol (Westang Biotech Co. Ltd, Shanghai, China). The sensitivities of the kits were 4 pg/ml for IL-6, 8 pg/ml for IL-1*β*, 15 pg/ml for IL-17A, and 17 pg/ml for TNF-*α*.

### Statistical analyses

Data are expressed as mean values ± standard errors (SEs). Survival rate of mice was analyzed by the Log-rank (Mantel-Cox) test. Multiple comparisons were performed using one way analysis of variance (ANOVA), followed by Fisher protected least significant difference test. Differences were considered statistically significant at P < 0.05.

## Additional Information

**How to cite this article**: Li-Sha, G. *et al.* Dose-dependent protective effect of nicotine in a murine model of viral myocarditis induced by coxsackievirus B3. *Sci. Rep.* **5**, 15895; doi: 10.1038/srep15895 (2015).

## Supplementary Material

Supplementary Information

## Figures and Tables

**Table 1:** Effects of nicotine on myocardial histopathology at days 7 and 14 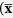 ± e).

Group	N	Infiltration		Necrosis	
		7 day	14 day	7 day	14 day
Normal	8	ND	ND	ND	ND
Myocarditis	8	2.63 ± 0.26	1.75 ± 0.25	2.00 ± 0.33	2.5 ± 0.38
0.1 mg/kg nicotine	8	2.13 ± 0.13	1.50 ± 0.19	1.50 ± 0.26	2.25 ± 0.25
0.2 mg/kg nicotine	8	1.50 ± 0.19*	1.13 ± 0.23	1.13 ± 0.29*	1.30 ± 0.18**
0.4 mg/kg nicotine	8	1.38 ± 0.18*	1.00 ± 0.27*	1.00 ± 0.31*	1.25 ± 0.16**

ND, not detected.

*P < 0.05, **P < 0.01 versus myocarditis.

**Table 2:** Primer sequences used for real-time PCR.

RNA	Primers	
CVB3	F-GTCTGCCTGCGTTTATTTC	R-ACTCAGCGTATCGTTTGGA
IL-17	F-TTTAACTCCCTTGGCGCAAAA	R-CTTTCCCTCCGCATTGACAC
IL-6	F-TGCCTTCTTGGGACTGAT	R-TAAGCCTCCGACTTGTGA
TNF-*α*	F-CACGCTCTTCTGTCTACTGA	R-AGGTACAACCCATCGGCTG
IL-1*β*	F-CCTTCTTTTCCTTCATCTTT	R-CGTTGCTTGGTTCTCCTTGT
GAPDH	F-AGGGAAATCGTGCGTGACAT	R-CATCTGCTGGAAGGTGGACA
