# Erratum: Dose-dependent protective effect of nicotine in a murine model of viral myocarditis induced by coxsackievirus B3

**DOI:** 10.1038/srep17247

**Published:** 2015-12-14

**Authors:** Ge Li-Sha, Zhao Jing-Lin, Chen Guang-Yi, Liu Li, Zhou De-Pu, Li Yue-Chun

Scientific Reports 5: Article number: 1589510.1038/srep15895; published online: 10282015; updated: 12142015

In the original HTML version of this Article, Li Yue-Chun was incorrectly listed as being affiliated with ‘Department of Pediatrics, Second Affiliated Hospital of Wenzhou Medical University, Wenzhou 325000, China’. The correct affiliation is listed below:

Department of Cardiology, Second Affiliated Hospital of Wenzhou Medical University, Wenzhou 325000, China

This has now been corrected.

