# Supplementary material for: Dose-dependent protective effect of nicotine in a murine model of viral myocarditis induced by coxsackievirus B3
Source: Sci Rep. 2015 Oct 28;5:15895. doi: 10.1038/srep15895 (PMC4623743; doi:10.1038/srep15895)
Supplement: Supplementary Information [file srep15895-s1.pdf]

Dose-dependent protective effect of nicotine in a murine model of viral myocarditis induced by coxsackievirus B3

Ge Li-Sha [a †] MD, Zhao Jing-Lin [b †] MD, Chen Guang-Yi [b] MD, Liu Li [b] MD, Zhou De-Pu [b] MD, Li Yue-Chun [b *] MD

Experiment 1

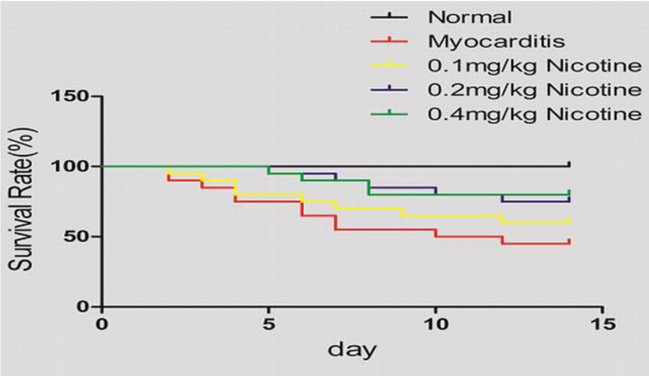

Experiment 2

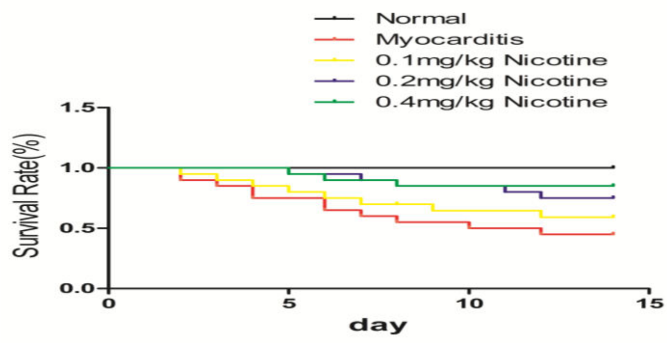

Experiment 3

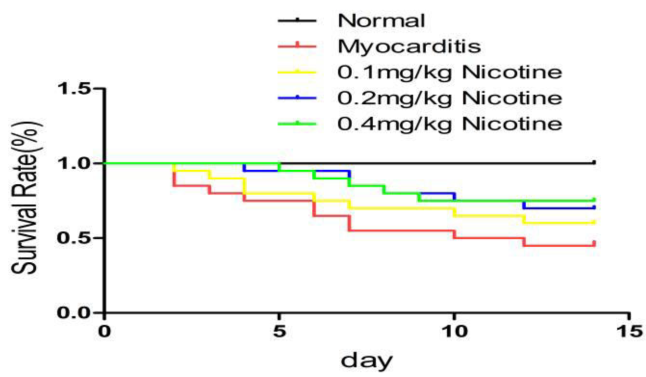

Survival rate	Experiment 1	Experiment 2	Experiment 3	P
Myocarditis	45%	40%	40%	>0.05
0.1mg/kg Nicotine	55%	50%	50%	>0.05
0.2mg/kg Nicotine	75%	75%	70%	>0.05
0.4mg/kg Nicotine	80%	85%	75%	>0.05

Additional Figure 1. Comparsion of survival rates among the 3 experiments. No significant difference was found among the 3 experiments.

Experiment 1

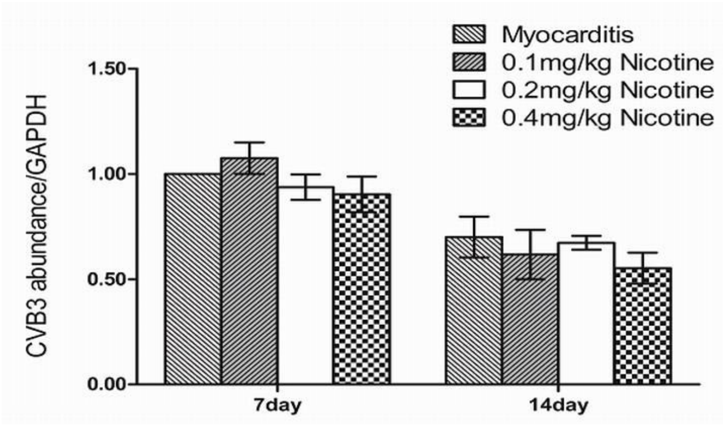

Experiment 2

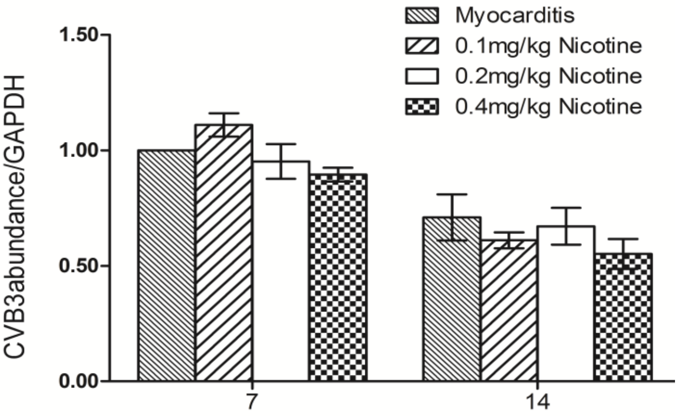

Experiment 3

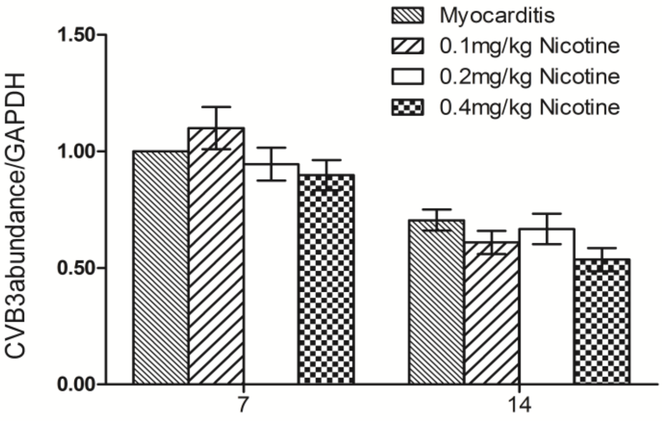

7 day	Experiment 1	Experiment 2	Experiment 3	P

Myocarditis	1.000±0.00	1.000±0.00	1.000±0.00	＞0.05
0.1mg/kg Nicotine	1.080±0.16	1.110±0.10	1.100±0.18	＞0.05
0.2mg/kg Nicotine	0.955±0.15	0.952±0.15	0.945±0.14	＞0.05
0.4mg/kg Nicotine	0.907±0.15	0.895±0.06	0.898±0.13	＞0.05

14 day	Experiment 1	Experiment 2	Experiment 3	P
Myocarditis	0.703±0.18	0.710±0.20	0.705±0.09	＞0.05
0.1mg/kg Nicotine	0.610±0.20	0.611±0.07	0.609±0.10	＞0.05
0.2mg/kg Nicotine	0.660±0.06	0.671±0.16	0.667±0.13	＞0.05
0.4mg/kg Nicotine	0.527±0.15	0.552±0.13	0.536±0.10	＞0.05

Additional Figure 2. Comparsion of CVB3 mRNAs among the 3 experiments. No significant difference was found among the 3 experiments.

Experiment 1

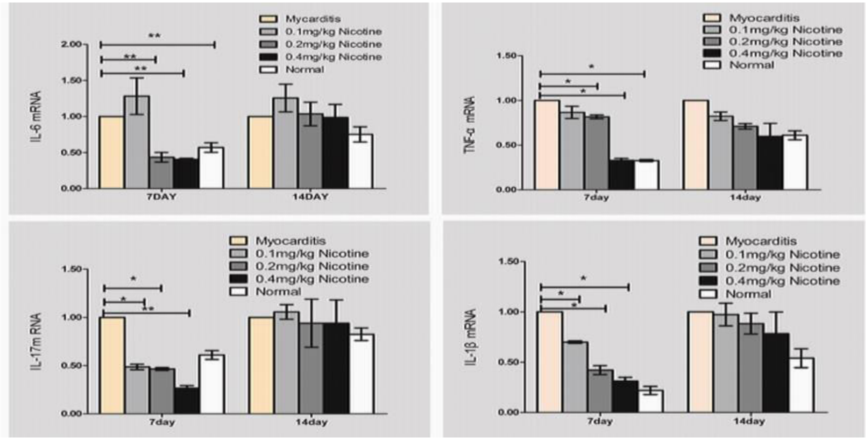

Experiment 2

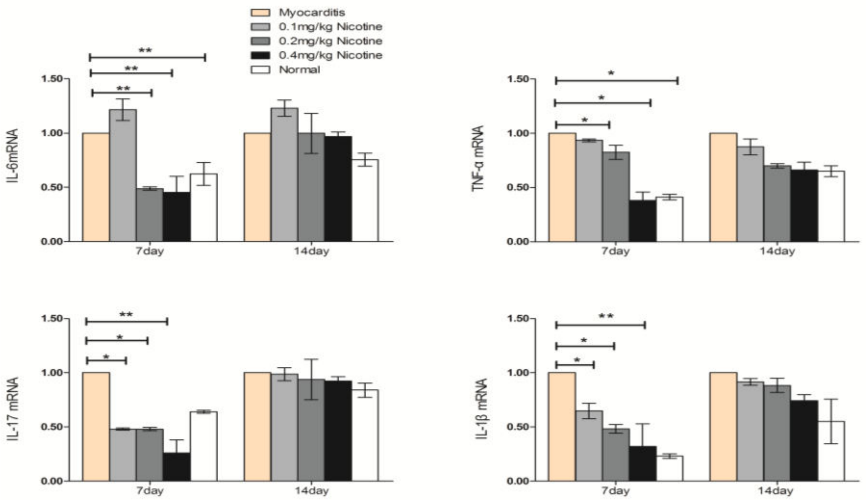

Experiment 3

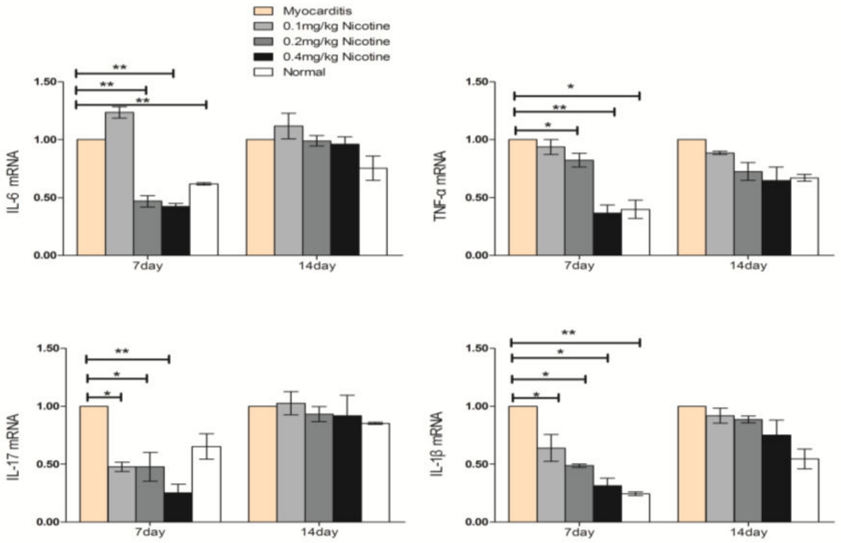

Normal group

7 day	Experiment 1	Experiment 2	Experiment 3	P
IL-6	0.613±0.08	0.625±0.21	0.618±0.12	>0.05
TNF-α	0.400±0.02	0.412±0.05	0.399±0.16	>0.05
IL-1β	0.230±0.10	0.230±0.04	0.245±0.03	>0.05
IL-17	0.655±0.12	0.640±0.03	0.652±0.22	>0.05
14 day				
IL-6	0.751±0.13	0.755±0.12	0.754±0.21	>0.05
TNF-α	0.678±0.08	0.650±0.10	0.670±0.06	>0.05
IL-1β	0.555±0.20	0.550±0.41	0.545±0.17	>0.05
IL-17	0.855±0.14	0.840±0.13	0.852±0.02	>0.05

0.1mg/kg Nicotine group

7 day	Experiment 1	Experiment 2	Experiment 3	P
IL-6	1.250±0.43	1.215±0.20	1.235±0.10	>0.05
TNF-α	0.930±0.16	0.935±0.03	0.937±0.13	>0.05
IL-1β	0.645±0.02	0.647±0.14	0.640±0.23	>0.05
IL-17	0.470±0.08	0.480±0.02	0.477±0.08	>0.05
14 day				
IL-6	1.236±0.12	1.230±0.15	1.118±0.22	>0.05
TNF-α	0.892±0.13	0.875±0.15	0.886±0.03	>0.05
IL-1β	0.978±0.22	0.915±0.06	0.918±0.13	>0.05
IL-17	1.057±0.18	0.987±0.12	1.027±0.20	>0.05

0.2mg/kg Nicotine group

7 day	Experiment 1	Experiment 2	Experiment 3	P
IL-6	0.472±0.12	0.488±0.03	0.468±0.10	>0.05
TNF-α	0.817±0.04	0.825±0.13	0.823±0.12	>0.05
IL-1β	0.431±0.10	0.483±0.08	0.487±0.03	>0.05
IL-17	0.454±0.02	0.480±0.22	0.478±0.25	>0.05
14 day				
IL-6	1.001±0.12	0.998±0.37	0.990±0.09	>0.05
TNF-α	0.757±0.10	0.700±0.04	0.725±0.15	>0.05
IL-1β	0.886±0.22	0.883±0.13	0.885±0.06	>0.05
IL-17	0.930±0.45	0.938±0.37	0.932±0.13	>0.05

0.4mg/kg Nicotine group

7 day	Experiment 1	Experiment 2	Experiment 3	P
IL-6	0.418±0.07	0.453±0.30	0.425±0.05	>0.05
TNF-α	0.370±0.05	0.380±0.16	0.365±0.14	>0.05
IL-1β	0.318±0.08	0.320±0.42	0.313±0.13	>0.05
IL-17	0.255±0.10	0.260±0.24	0.253±0.15	>0.05
14day				
IL-6	0.951±0.17	0.969±0.08	0.960±0.13	>0.05

TNF-α	0.658±0.32	0.660±0.15	0.648±0.23	>0.05
IL-1β	0.754±0.41	0.743±0.11	0.750±0.26	>0.05
IL-17	0.925±0.43	0.923±0.08	0.920±0.35	>0.05

Additional Figure 3. Comparsion of cytokine mRNAs among the 3 experiments. No significant difference was found among the 3 experiments.

Experiment 1

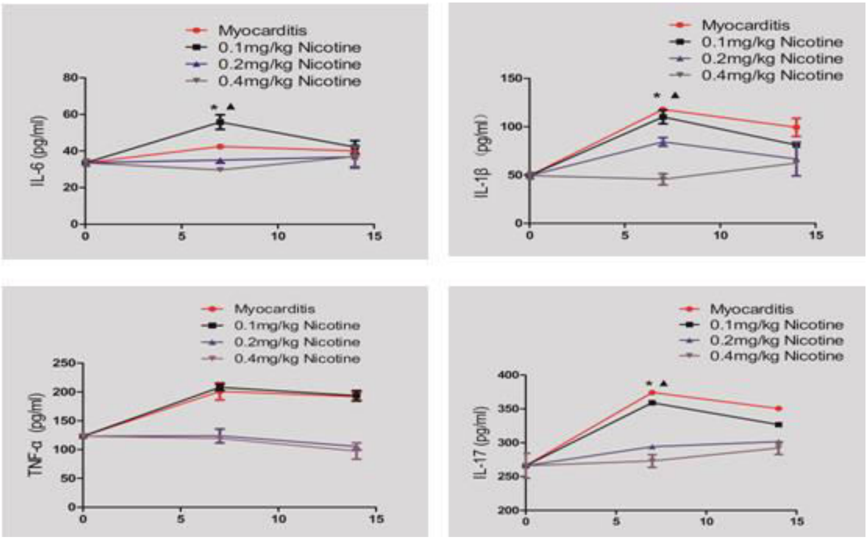

Experiment 2

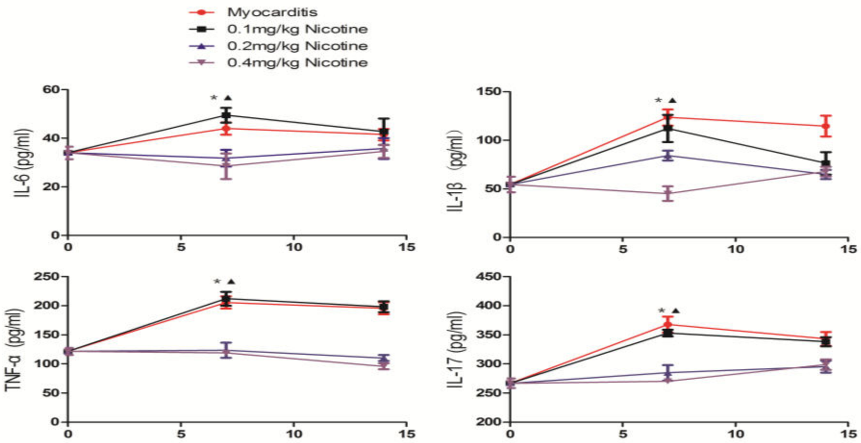

Experiment 3

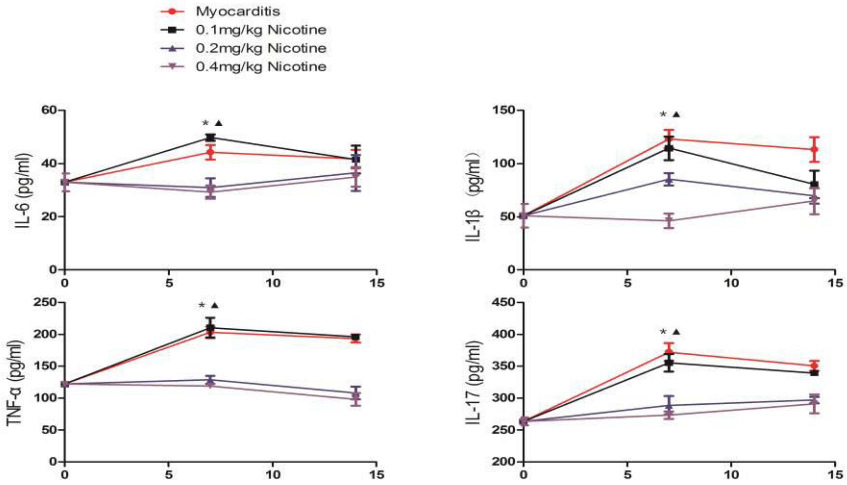

Myocarditis group (pg/ml)

7day	Experiment 1	Experiment 2	Experiment 3	P
IL-6	44.75±0.10	44.00±2.58	44.25±2.75	>0.05
TNF-α	201.00±15.00	205.60±5.91	203.25±8.92	>0.05
IL-1β	123.50±2.34	123.50±8.26	123.25±8.59	>0.05
IL-17	370.00±3.34	367.50±13.69	372.25±14.03	>0.05
14day				
IL-6	40.25±2.62	41.50±2.38	41.75±3.40	>0.05
TNF-α	191.75±7.66	195.50±10.34	193.50±6.61	>0.05
IL-1β	100.00±8.89	114.50±10.78	113.25±11.52	>0.05
IL-17	347.00±2.25	343.00±11.88	350.50±7.76	>0.05

0.1mg/kg Nicotine group (pg/ml)

7day	Experiment 1	Experiment 2	Experiment 3	P
IL-6	50.25±2.87	49.50±3.10	49.75±1.26	>0.05
TNF-α	208.00±5.71	211.75±11.89	210.50±15.54	>0.05
IL-1β	113.00±8.90	112.25±13.93	114.50±11.03	>0.05
IL-17	351.50±4.34	353.00±5.88	355.50±13.67	>0.05
14day				
IL-6	40.87±2.96	42.75±5.43	41.50±5.25	>0.05
TNF-α	193.50±8.70	198.00±9.41	196.00±4.32	>0.05
IL-1β	78.89±2.23	76.25±11.25	80.50±12.79	>0.05
IL-17	320.98±5.97	338.00±7.39	339.50±0.57	>0.05

0.2mg/kg Nicotine group (pg/ml)

7day	Experiment 1	Experiment 2	Experiment 3	P
IL-6	30.25±1.0	31.75±3.40	31.00±3.56	>0.05
TNF-α	123.76±12.21	123.50±13.02	128.75±6.34	>0.05
IL-1β	81.75±4.85	84.25±5.12	85.25±15.26	>0.05
IL-17	290.00±4.20	284.75±12.00	288.50±14.93	>0.05
14day				
IL-6	37.50±4.56	35.75±4.27	36.50±6.74	>0.05
TNF-α	105.50±6.13	110.50±5.19	108.25±10.04	>0.05
IL-1β	66.34±4.89	64.75±4.78	69.75±7.13	>0.05
IL-17	296.75±2.23	295.25±10.17	297.00±4.96	>0.05

0.4mg/kg Nicotine group (pg/ml)

7day	Experiment 1	Experiment 2	Experiment 3	P
IL-6	28.75±0.08	28.50±5.29	29.25±2.50	>0.05
TNF-α	119.50±3.19	118.75±3.59	119.00±3.74	>0.05
IL-1β	49.00±5.23	45.00±7.52	46.25±6.70	>0.05
IL-17	269.25±8.61	270.25±4.19	273.50±6.02	>0.05
14day				
IL-6	37.25±3.09	34.50±2.64	35.00±3.74	>0.05

TNF-α	97.50±14.00	96.25±5.12	98.25±10.01	>0.05
IL-1β	65.55±12.21	67.75±5.12	64.75±12.41	>0.05
IL-17	290.00±14.89	298.75±8.77	291.00±14.76	>0.05

Additional Figure 4. Comparsion of cytokine proteins among the 3 experiments. No significant difference was found among the 3 experiments.
